# A threefold approach to rescue the 2030 Agenda from failing

**DOI:** 10.1093/nsr/nwad015

**Published:** 2023-01-12

**Authors:** Prajal Pradhan

**Affiliations:** Potsdam Institute for Climate Impact Research (PIK), Member of the Leibniz Association, Germany; Bauhaus Earth GmbH, Germany

## Abstract

Rescuing the 2030 Agenda for Sustainable Development from failing requires prioritizing Sustainable Development Goals (SDGs), understanding the impacts of underachieving SDGs, and building a post-2030 Agenda based on scientific evidence.

Countries are not on track to meet the 2030 Agenda for Sustainable Development [1] that calls for transformative changes to shift the world onto a sustainable and resilient path. The 2030 Agenda comprises 17 Sustainable Development Goals (SDGs) and 169 targets to be achieved by 2030, balancing the three sustainability dimensions—social, economic, and environmental. So far, SDGs have had a limited transformative impact [2] because of their selective implementation without considering their complex interactions. SDGs interact positively (i.e. synergies) or negatively (i.e. trade-offs) depending on context- and location-specific mechanisms. Failing to meet SDGs will negatively affect the lives of billions of people and worsen socioeconomic and environmental crises. The COVID pandemic has decelerated or reversed the process of the 2030 Agenda. Therefore, the next few years are crucial to accelerate SDG progress and adopt a post-2030 Agenda or a follow-up of SDGs. This perspective proposes three research avenues with a threefold scientific approach to address these urgent needs (Fig. 1).

**Figure 1.**
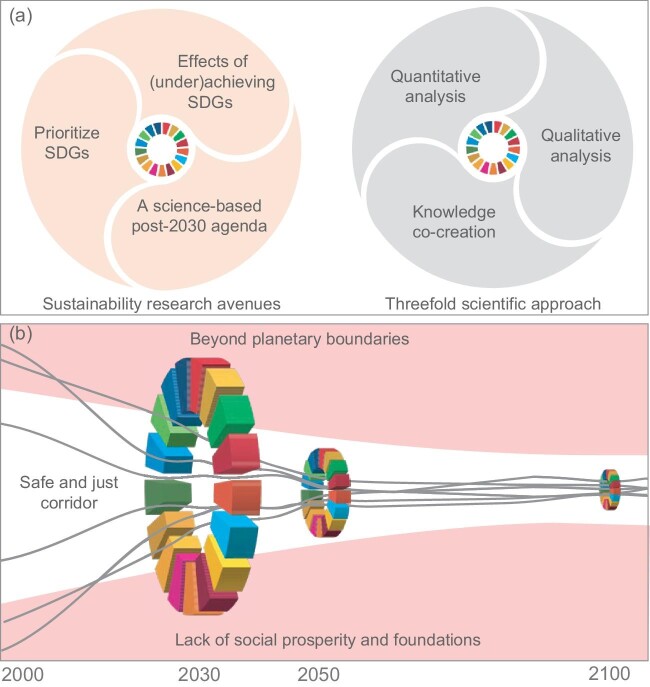
A conceptual framework for ensuring sustainability beyond achieving Sustainable Development Goals (SDGs). (a) The threefold scientific approach (left) combines three methods to rescue the 2030 Agenda from failing (right). (b) Sustainability requires building social prosperity and foundations without transgressing planetary boundaries (the red areas), i.e. within the safe and just corridor for people and the planet (the white area). SDGs are a waypoint for sustainability. The safe and just corridor could narrow down over time due to the need to sustain an increased population with changing lifestyles. Grey lines illustrate sustainable development pathways of various countries, referring to the need for transformative change.

The research avenues comprise systematic prioritization of SDGs instead of cherry-picking, understanding linkages between SDGs and sustainability, and co-creating a science-based post-2030 Agenda (Fig. 1a, left).

## Prioritizing SDGs.

Rescuing the 2030 Agenda requires prioritizing SDGs where transformative actions can maximize synergies and resolve trade-offs, leading to progress across most SDGs. Systematic SDG interaction analysis, including their networks and underlying mechanisms, can help prioritize SDGs [3,4]. Although an increasing number of studies apply a broad range of qualitative, quantitative, and mixed methods to investigate SDG interactions, insights into diverse mechanisms underlying these interactions are limited [2]. These insights are crucial to understanding what enables or disables transformative changes. Thus, the first research avenue is to systemically prioritize SDGs by identifying their complex interactions and underlying mechanisms, going beyond the state-of-the-art. Such prioritization will help to effectively invest limited resources in order to speed up progress across all SDGs at a systemic level, which is not achievable by cherry-picking.

## SDGs and sustainability.

A reason for the limited efforts to achieve SDGs is a lack of understanding of the full effects of underachieving SDGs on sustainability. Ensuring sustainability requires building social prosperity and foundations within planetary boundaries, i.e. a safe and just corridor for people and the planet [5] (Fig. 1b). Transgressing the planetary boundaries destabilizes Earth's systems [5]. Social prosperity (i.e. economic activities) needs to generate social foundations that comprise people's basic needs (e.g. food, water, health, political voice, social equity, and gender equality) [6]. Therefore, the second research avenue is to investigate the effects of (under)achieving SDGs on social prosperity and foundations and planetary boundaries. This investigation will recognize how underachieving SDGs will fail to shift the world onto a sustainable and resilient path. It will also highlight the required extra efforts, besides meeting SDGs, to ensure sustainability beyond 2030.

## Science-based post-2030 Agenda.

In 2030, another global agenda will replace SDGs, and this political process might start soon. Scientific communities need urgent preparation to make this post-2030 Agenda more science-based, addressing the criticisms of SDGs. These criticisms include SDGs being more aspirational than quantified or SMART (specific, measurable, achievable, realistic, and time-bounded) [7]. Thus, the third research avenue is to co-create a framework for a science-based post-2030 Agenda by combining lessons from SDGs and state-of-the-art science with stakeholders’ knowledge.

The three research avenues could be explored by applying a threefold scientific approach elaborated below (Fig. 1a, right).

## Quantitative analysis.

Understanding SDG interactions and (under)achieving SDG effects can be generated by applying various quantitative analyses on empirical and modelled data. For SDG interactions, a challenge is to identify suitable quantitative methods to discover causal relations, going beyond their statistical associations. Due to data limitations and the multi-dimensionality of SDGs, quantitative analyses need to be based on data from multiple sources. For example, the unified SDG database [8] combines the SDG data from the United Nations Statistics Division with the World Bank and the Sustainable Development Solutions Network. Still, closing the SDG data gap is crucial to monitoring and reviewing the achievement of the 2030 Agenda. For modelled data, most integrated assessment models (IAMs) currently developed to study energy, economy, climate, and land interactions have poor social SDG coverage [9]. Therefore, quantitative analyses need to include various IAMs’ outcomes and projections based on other models to fully understand the effects of (under)achieving SDGs. A comparable analysis of multiple model outcomes requires developing a standard protocol and initiating an SDG model intercomparison and development (SDGMID) process.

## Qualitative analysis.

Transformative actions to achieve SDGs and ensure sustainability must be based on the best available scientific evidence. Although quantitative analyses can identify SDG interactions, further qualitative investigations are required to understand the underlying mechanisms or processes. Similarly, dealing with limited data requires alternative methods to quantitative analyses. These methods include meta-analyses of the literature, expert elicitations, and qualitative literature analysis. For example, systematic literature analysis can synthesize evidence [10] on SDG interactions’ mechanisms and the effects of (under)achieving SDGs, including new insights on the most pressing global challenges. However, there might be two limitations: statistical noises due to studies’ context- and location-specific dependencies and limited literature on specific topics related to SDGs and sustainability. Expert elicitations can address these limitations by filtering the noises and filling the gaps in the literature. Additionally, quantitative methods can be applied to further investigate generated qualitative data, leading to a mixed-method approach.

## Knowledge co-creation.

For transformative changes, knowledge co-creation with stakeholders is also essential. It will help fill the gaps in data and literature and provide context- and location-specific insights, supporting the localization of SDGs to implement the 2030 Agenda at the local level. Further, stakeholders will share ownership of co-created knowledge. This ownership is crucial for implementing transformative actions to achieve SDGs and co-creating a science-based post-2030 Agenda. Various stakeholders must be engaged during the knowledge co-creation process and SDG localization. They include the public sector, the private sector, academia, (inter)national and non-governmental organizations, and the general public. It is essential to consider diversity, inclusion, and gender dimensions during stakeholder engagements for a balanced representation. Various methods for stakeholder engagement include surveys, workshops, focus groups, webinars, and interviews.

In conclusion, addressing the three research avenues can rescue the 2030 Agenda from failing by generating three novel understandings. The first is understanding mechanisms underlying SDG interactions and transformative actions to resolve the current trade-offs among SDGs, resulting in win-win solutions. The second is understanding the potential and limitations of SDGs in shifting the world towards a sustainable and resilient path. The third is to initiate a process to co-create a science-based post-2030 Agenda for sustainable transformation for building social prosperity and foundations within planetary boundaries. These understandings are crucial for implementing adequate actions to accelerate SDG progress during the post-COVID recovery. Generating these understandings requires combining quantitative and qualitative analyses with knowledge co-creation.
